# Life-Threatening Internal Hernia Through an Omental Band With Closed-Loop Obstruction in a Virgin Abdomen and Normal Laboratory Findings: A Case Report

**DOI:** 10.7759/cureus.106823

**Published:** 2026-04-10

**Authors:** Shooq Faqeeh, Mahdi Zamiri Oskouei

**Affiliations:** 1 General Surgery, Dubai Health Authority, Dubai, ARE; 2 General Surgery, Health and Medical Services (HMS) Al Garhoud Hospital, Dubai, ARE

**Keywords:** abdominal imaging, bowel resection, internal hernia, intestinal obstruction, small bowel obstruction, surgical anastomosis, surgical management, transomental hernia, virgin abdomen

## Abstract

Small bowel obstruction (SBO) is a surgical emergency commonly caused by adhesions from prior abdominal surgeries or hernias. In contrast, SBO caused by omental bands with no history of trauma or abdominal surgeries is rare, and only a few cases have been reported. We present a case of a previously healthy 44-year-old male who presented with sudden, severe generalized colicky abdominal pain after eating, associated with nausea, vomiting, and abdominal tenderness, without distension. Initial lab results and X-ray findings were unremarkable. Intravenous contrast computed tomography (CT) scan revealed closed-loop jejunal obstruction with irreversible ischemic changes. Emergency intra-operative findings indicated mechanical obstruction caused by an omental band, which led to bowel necrosis. Laparoscopic necrotic bowel resection and anastomosis were performed, and the patient recovered uneventfully. The lack of specific signs and symptoms associated with internal hernias (IHs) poses a tremendous diagnostic challenge, with CT scans playing a pivotal role in guiding management.

## Introduction

An internal hernia (IH) refers to the protrusion of a viscus, most often the small intestine, through a normal or pathological peritoneal or mesenteric defect within the abdominal or pelvic cavity. IHs account for approximately 0.6-5.8% of all small bowel obstruction cases [1]. While IH may be secondary to congenital mesenteric defects [2], most instances are associated with iatrogenic or post-traumatic factors, along with other conditions related to peritoneal inflammation. They typically present as acute small bowel strangulation. In patients without a prior surgical history, spontaneous IH may arise due to age-related omental atrophy [3]. Nevertheless, identifying an IH in a patient with a virgin abdomen remains exceptionally rare [4]. IH, though rare, is associated with significant morbidity and mortality rates exceeding 50%, underscoring the importance of early recognition and prompt surgical intervention [5]. We report a rare case of spontaneous IH presenting as small bowel obstruction (SBO) in a patient with no prior abdominal surgery, which was successfully diagnosed and managed in our hospital.

## Case presentation

A 44-year-old male with no known medical illnesses presented to the emergency department with acute symptoms of generalized abdominal pain 30 min after eating, associated with nausea and vomiting. The abdominal pain was colicky in nature, and the pain score was graded as 9 out of 10. He had never experienced a similar presentation before. No exacerbating or alleviating factors were noted. The patient denied any preceding illness or trauma. No other constitutional symptoms, bowel or urinary habit changes were noted. Past medical, surgical, and social history were unremarkable, with no family history of malignancies. Initial physical evaluation showed a vitally stable, alert, and oriented patient lying uncomfortably in bed. Abdominal examination revealed generalized abdominal tenderness, without distension, guarding, or rigidity. Bowel sounds were decreased. The hernial orifices were intact, and digital rectal examination findings were unremarkable.

**Figure 1:**
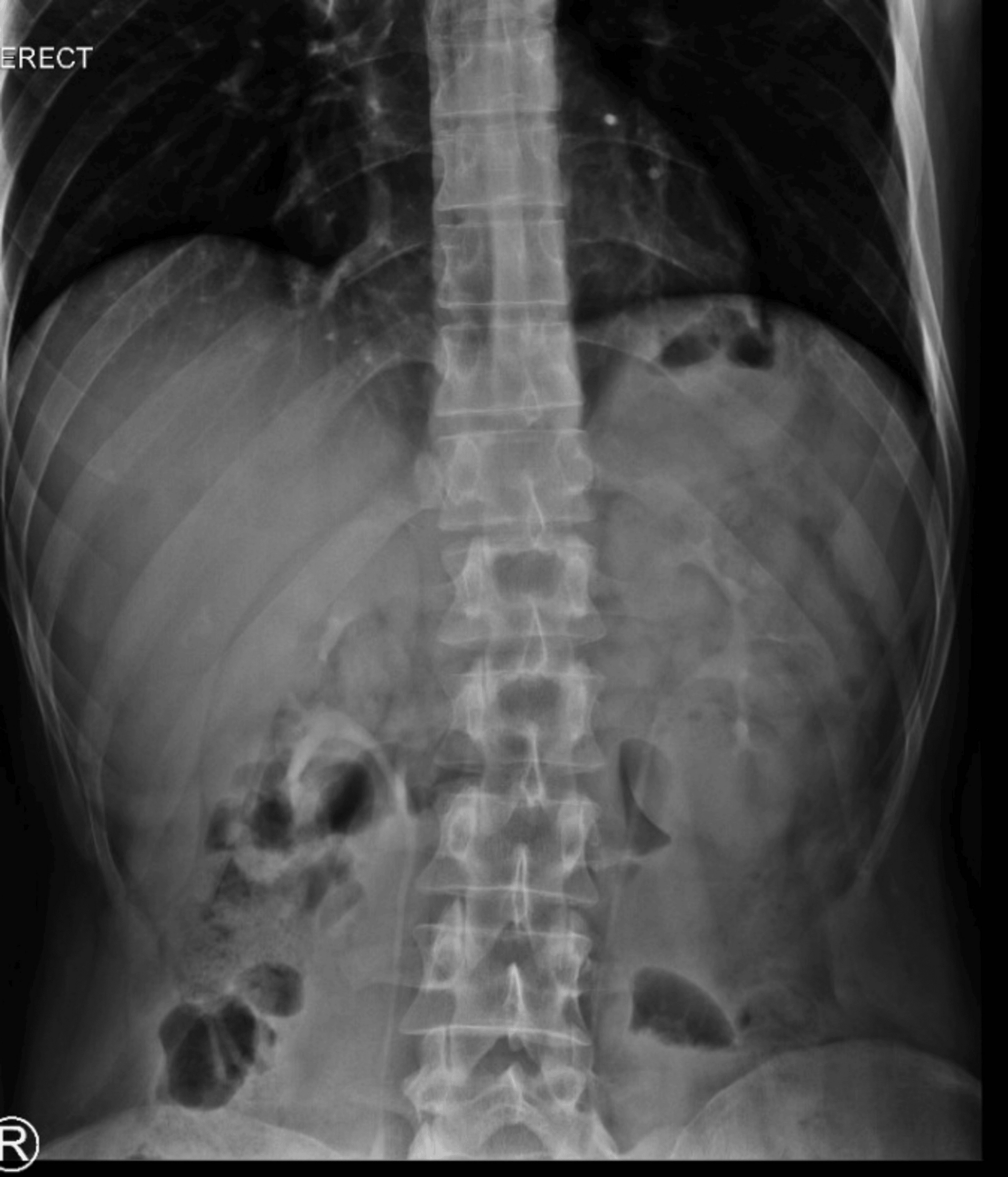
A plain film of the abdomen showing usual gas pattern.

Laboratory findings indicated serum lactate dehydrogenase (LDH) of 111 U/L (normal range: 125-243 U/L), potassium level of 3.3 mmol/L (normal range: 3.5-5.5 mmol/L), bicarbonate level of 20 mmol/L (normal range: 22-30 mmol/L), normal white blood cell count, hemoglobin, blood urea nitrogen, and C-reactive protein. Plain abdominal X-ray in the upright position showed no air/fluid levels and no air under the domes of the diaphragm (Figure 1).

**Figure 2:**
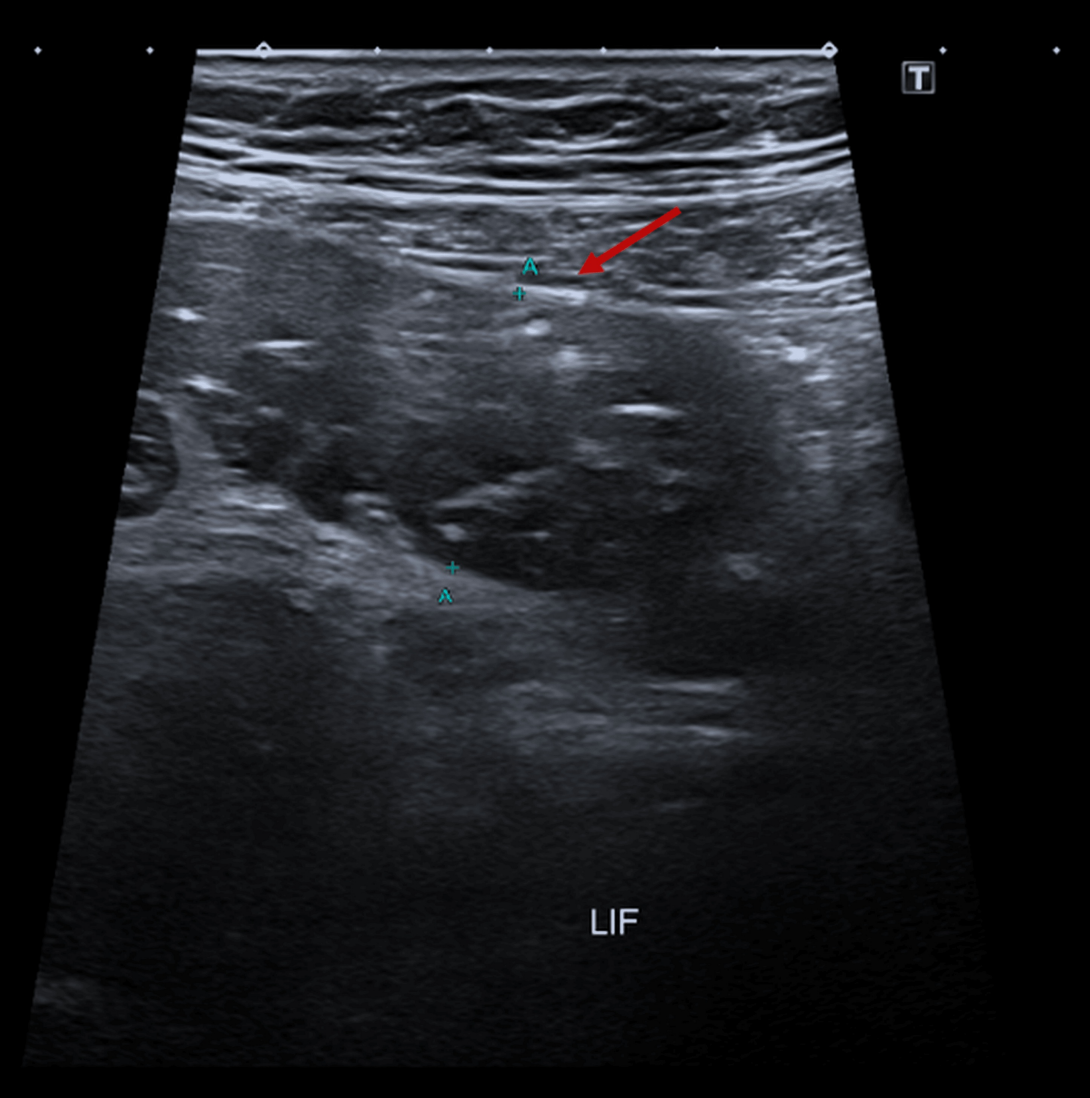
Ultrasound scan of abdomen and pelvis revealing mildly dilated intestinal loops (arrow) measuring around 2.5 cm.

**Figure 3:**
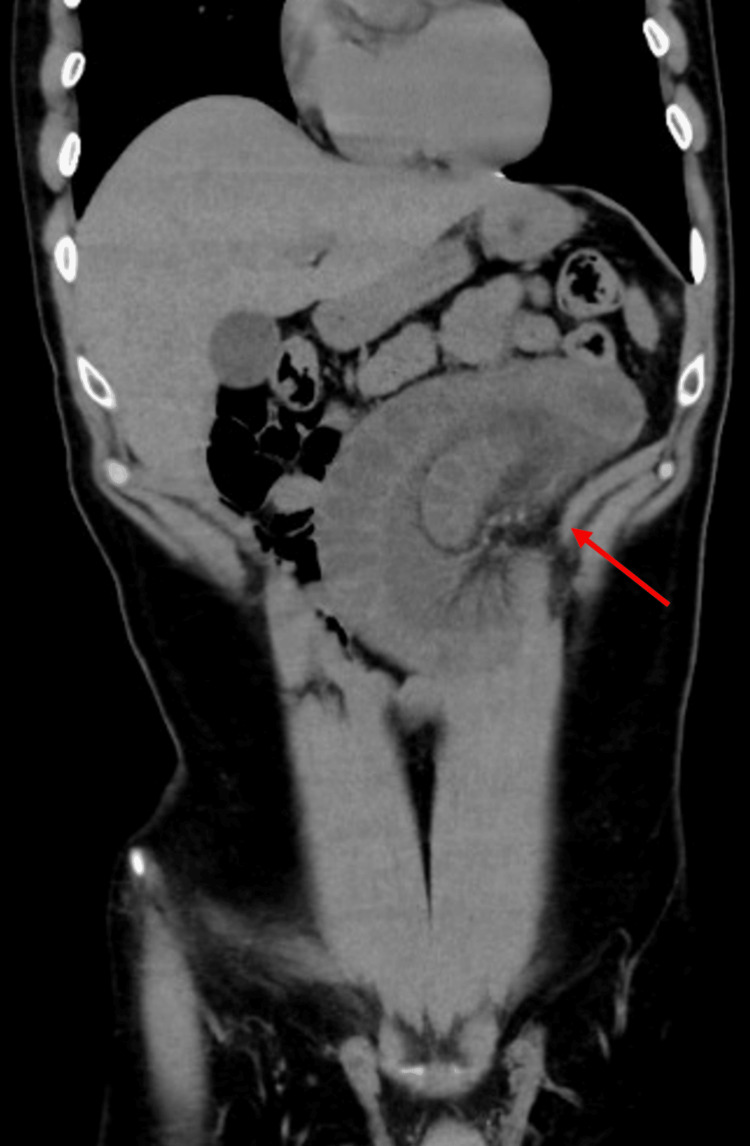
A contrast-enhanced CT scan of the abdomen and pelvis reveals a cluster of hypo-enhancing jejunal loops (indicated by arrow) measuring about 3 cm, with mild diffuse mural thickness.

Ultrasound scan of the abdomen and pelvis revealed mildly dilated intestinal loops in the left lower abdomen and diffuse abdominal gaseous distension, prompting further investigation (Figure 2). A pre-contrast CT scan was performed, followed by a triphasic post-IV contrast scan, which revealed unanticipated findings of clustered jejunal C-shaped loops in the left upper abdomen with linear vascular engorgement and contained fluid, converging towards a beak-like luminal narrowing. The hypo-enhancing intestinal loops showed diffuse mural thickening suggestive of mural edema and ischemia, along with a minimal amount of free fluid in the right sub-diaphragmatic perihepatic, right paracolic gutter, and pelvic cavity (Figure 3). The patient was admitted for an emergent exploratory laparoscopy.

**Figure 4:**
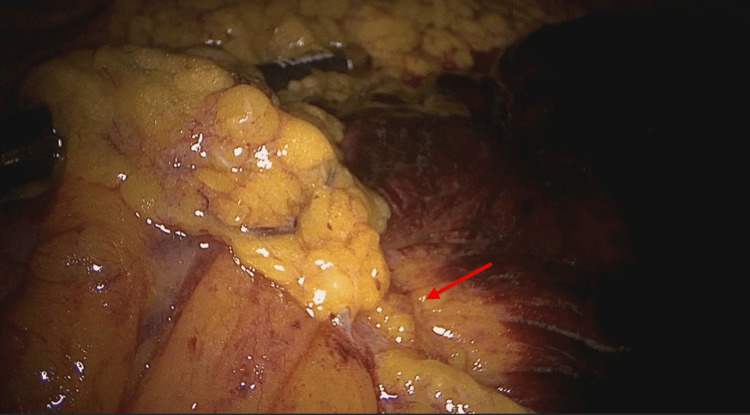
The origin point of attachment (arrow) between the omental band and the mesentery.

**Figure 5:**
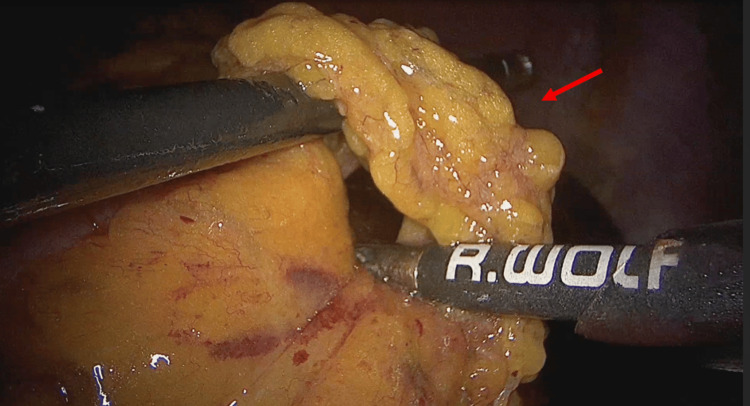
Herniating bowel (arrow) through the omental band being released.

**Figure 6:**
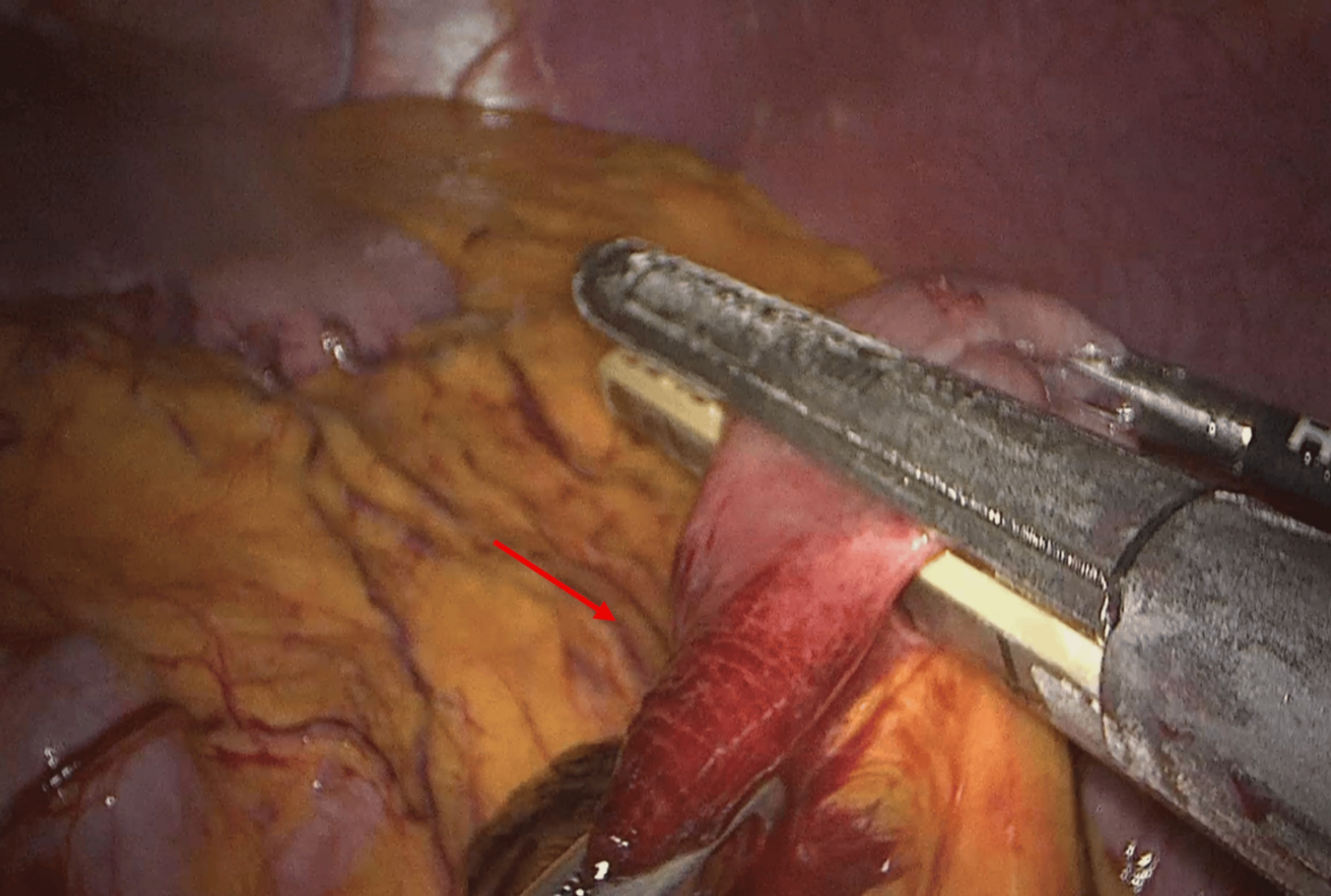
Congested bowel loops along with the point of transition (arrow) between the affected and unaffected bowel segments.

**Figure 7:**
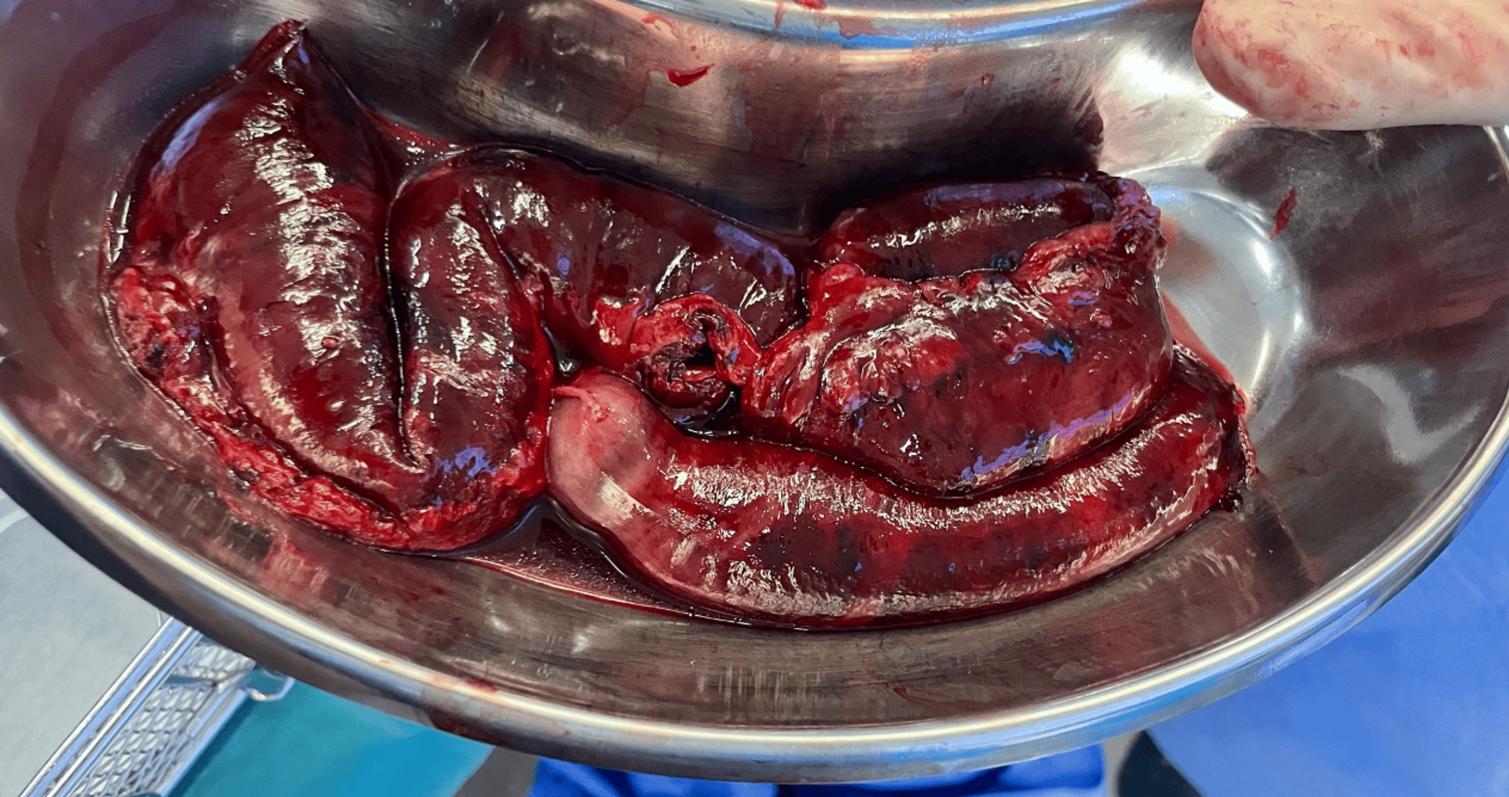
Post-operative resection of the necrotic bowel segment measuring approximately 50 cm. Despite extensive ischemia, LDH levels remained low, highlighting the potential unreliability of LDH as a marker. LDH: lactate dehydrogenase

The intra-operative findings confirmed the diagnosis of internal herniation by an omental band leading to irreversible jejunal ischemia. The arrow in Figure 4 demonstrates the origin point of attachment between the omentum and the mesentery. The omental band was divided, releasing the obstruction in the affected bowel (Figure 5). Figure 6 demonstrates the transition point between the affected and unaffected bowel segments. Laparoscopic resection of the necrotic bowel segment with side-to-side primary anastomosis and closure of the mesenteric defect was performed (Figure 6). Approximately 50 cm of bowel was affected (Figure 7). The patient recovered uneventfully, and the symptoms were resolved. The patient was discharged after six days. Histology of the resected specimen revealed necrosis and hemorrhagic slough in the mucosa.

## Discussion

IH occurs when the viscera protrude through a mesenteric or peritoneal opening, but the herniated viscera stay inside the abdominal cavity [6]. The paraduodenal (53%) and pericecal (13%) hernias are the most common, followed by those through the foramen of Winslow (8%), the transmesenteric (2%), and transomental (1%) [7].

The majority of IH cases involve small bowel loops and are caused by either congenital or acquired defects in the attachment of various peritoneal folds [7]. Furthermore, operations such as gastrectomy, choledochojejunostomy, and Roux-en-Y gastric bypass are associated with a higher risk of developing an IH [4,8]. The presentation of our patient does not fit any of the frequently listed causes of IH. Our patient was a young adult without a history of trauma, previous abdominal surgery, or comorbidities that would predispose to omental weakness, whereas most cases are ascribed to iatrogenic or post-traumatic causes or senile omental atrophy in the elderly [9]. It is less common to find an IH in other cases of a virgin abdomen.

As the clinical symptoms of an IH are non-specific and resemble those of other causes of acute bowel obstruction, such as abdominal pain, nausea, vomiting, abdominal distention, and constipation, pre-operative diagnosis of this condition is frequently difficult and raises the possibility of a missed or delayed diagnosis [10,11]. Consequently, a high index of clinical suspicion is necessary, especially when patients exhibit small bowel obstruction symptoms without a known underlying cause. CT imaging played a crucial role in establishing a pre-operative diagnosis of IH and enabling prompt management, as demonstrated in our case.

Although CT imaging lacks a single pathognomonic feature for pre-operative internal hernia diagnosis, skilled radiologists can still use it effectively. When radiologic findings are combined, diagnostic accuracy improves, with greater sensitivity and specificity. Early surgical exploration is recommended when CT results are inconclusive but clinical suspicion persists [12]. Although standard abdominal and pelvic CT examinations in patients with suspected high-grade SBO have demonstrated diagnostic accuracies of more than 90%, low-grade or intermittent obstruction has been diagnosed less accurately, with a sensitivity of only 48-50% and a specificity of 94% [13]. Furthermore, multiphasic contrast-enhanced CT is recommended for patients with suspected closed-loop obstruction to assess bowel perfusion and viability. It improves ischemia detection and guides surgical management [14].

Surgical treatment entails reducing the herniated bowel, with resection if viability is compromised, and repairing the omental defect to prevent recurrence [1,15]. Due to advancements in surgical techniques, laparoscopy has emerged as a safe and minimally invasive method of managing IH in the absence of bowel necrosis or perforation, laparotomy was typically preferred when the assessment of bowel viability is challenging. However, there are no well-defined criteria for assessing bowel viability, particularly in the laparoscopic setting [16].

While previous research has suggested that serum lactate dehydrogenase (LDH) levels greater than 800 IU/L are strongly associated with bowel compromise and have a high predictive value for gangrene in intestinal obstruction (sensitivity 75%, specificity 94%), our findings contradict this observation [17]. Despite the presence of 50 cm of bowel necrosis, LDH levels were found to be paradoxically low, which raises a concern about whether normal LDH levels are explicable medically in the presence of bowel necrosis. Our case emphasizes the timely detection and decision for prompt surgery, as the decision of an early operative intervention guided safe management and prevented complications such as perforation.

## Conclusions

Bowel obstruction can be caused by a variety of factors, including rare etiologies such as IH, even in young patients who have not had abdominal surgery before. This case emphasizes the importance of keeping a high level of suspicion for unusual IH in patients with a virgin abdomen. Prompt recognition and surgical intervention are critical to avoid serious complications and reduce morbidity and mortality. Imaging, particularly CT, can help with pre-operative diagnosis, but definitive confirmation is often obtained during surgery. Our study contributes to the limited literature on IHs in young adults without predisposing factors, emphasizing the importance of a multidisciplinary approach for effective management.
